# Effect of Walking Adaptability on an Uneven Surface by a Stepping Pattern on Walking Activity After Stroke

**DOI:** 10.3389/fnhum.2021.762223

**Published:** 2022-01-04

**Authors:** Yusuke Sekiguchi, Keita Honda, Shin-Ichi Izumi

**Affiliations:** ^1^Department of Physical Medicine and Rehabilitation, Graduate School of Medicine, Tohoku University, Sendai, Japan; ^2^Department of Physical Medicine and Rehabilitation, Graduate School of Biomedical Engineering, Tohoku University, Sendai, Japan

**Keywords:** walking adaptability, walking activity, compensatory movement, stroke, uneven surface

## Abstract

Real-world walking activity is important for poststroke patients because it leads to their participation in the community and physical activity. Walking activity may be related to adaptability to different surface conditions of the ground. The purpose of this study was to clarify whether walking adaptability on an uneven surface by step is related to daily walking activity in patients after stroke. We involved 14 patients who had hemiparesis after stroke (age: 59.4 ± 8.9 years; post-onset duration: 70.7 ± 53.5 months) and 12 healthy controls (age: 59.5 ± 14.2 years). The poststroke patients were categorized as least limited community ambulators or unlimited ambulators. For the uneven surface, the study used an artificial grass surface (7 m long, 2-cm leaf length). The subjects repeated even surface walking and the uneven surface walking trials at least two times at a comfortable speed. We collected spatiotemporal and kinematic gait parameters on both the even and uneven surfaces using a three-dimensional motion analysis system. After we measured gait, the subjects wore an accelerometer around the waist for at least 4 days. We measured the number of steps per day using the accelerometer to evaluate walking activity. Differences in gait parameters between the even and uneven surfaces were calculated to determine how the subjects adapted to an uneven surface while walking. We examined the association between the difference in parameter measurements between the two surface properties and walking activity (number of steps per day). Walking activity significantly and positively correlated with the difference in paretic step length under the conditions of different surface properties in the poststroke patients (*r* = 0.65, *p* = 0.012) and step width in the healthy controls (*r* = 0.68, *p* = 0.015). The strategy of increasing the paretic step length, but not step width, on an uneven surface may lead to a larger base of support, which maintains stability during gait on an uneven surface in poststroke patients, resulting in an increased walking activity. Therefore, in poststroke patients, an increase in paretic step length during gait on an uneven surface might be more essential for improving walking activity.

## Introduction

Real-world walking activity is important for poststroke patients because it leads to their participation in the community and physical activity, decreasing their risks of mental illness, hypertension, hyperlipidemia, and diabetes (Ivey et al., 2005; Hafer-Macko, 2008; Barclay et al., 2015; Saunders et al., 2016). It is often measured as the number of steps per day by using an activity monitor. A systematic review demonstrated that the walking activity (ranging from 1,389 to 7,379 steps/day) in poststroke patients was lower than that (ranging from 6,294 to 14,730 steps/day) in age-matched healthy controls (English et al., 2014). Kono et al. (2015) demonstrated that the attainment of approximately > 6,000 steps/day may reduce the risk of new vascular events in poststroke patients. Therefore, the lack of real-world walking activity is a serious problem in patients with hemiparesis.

The factors associated with decreased physical activity, including walking activity, after stroke were age, sex, physical function (6-min walk test distance, comfortable gait speed, Berg balance scale, and cardiorespiratory fitness), depression, fatigue, self-efficacy, and quality of life (Thilarajah et al., 2018). Especially, the 6-min walk test was better able to discriminate real-world walking activity among home, limited community, and full community ambulators (Fulk et al., 2017). However, in a previous study, following training, changes in specific clinical walking measures, including 6-min walk test distance, explained up to 33% of the changes in walking activity, although those in gait biomechanics explained up to 86% of the variance in altered walking activity, with specific associations with kinematic parameters at hip joints in both lower limbs (Ardestani et al., 2019). Biomechanical assessment (i.e., lower limb kinematics during gait) may be more available than clinical walking measures for detecting changes in walking activity.

Walking adaptability is an essential requisite for achieving independent and safe community walking (Balasubramanian et al., 2014). A situation that requires walking adaptability in a real-life environment is walking on uneven surfaces that are neither flat or firm (Balasubramanian et al., 2014). Healthy controls decreased their step length and increased the height of their toe clearance and step time and width when walking on uneven surfaces (i.e., railroad ballast and artificial grass) (Allet et al., 2009; Menant et al., 2009; Wade et al., 2010). The shorter and slower steps were represented as a cautious gait pattern to decrease the risk of slipping (Wade et al., 2010). In patients with incomplete spinal injury, the step length decreased and the step time increased during walking on uneven surfaces (i.e., artificial grass, soft, and pebble surfaces), but the step length symmetry did not change, as in the healthy controls (Promkeaw et al., 2019). In contrast, adapting to uneven surfaces may be difficult for patients after the onset of cerebral injury because of the damaged supraspinal motor pathways (i.e., motor cortex, cerebral white matter, and/or internal capsule) that are critical for walking adaptability. Additionally, the motor modules, which organize muscle activities in the lower limbs during gait, were merged by the impaired nervous system due to stroke (Clark et al., 2010; Brough et al., 2018). The fewer motor modules resulted in more asymmetry propulsion [i.e., lower propulsion on the paretic side (PS) than on the non-PS] during gait in patients after stroke (Clark et al., 2010). Similarly, the merged plantarflexor motor module increased whole-body angular momentum at the frontal plane during gait in patients, representing decreased balance control (Brough et al., 2018). Therefore, it may be difficult to adapt to the uneven surface by merging plantarflexor modules due to the instability of patients with an impaired nervous system as in stroke. However, patients with cerebral palsy also adapted to uneven surfaces (i.e., polyurethane) with decreased gait speed and cadence, increased toe clearance, and increased knee and hip flexion and pelvic anterior tilt, similar to the healthy controls (Böhm et al., 2014). Although a previous study investigated walking on uneven surfaces in poststroke patients, the study only clarified increased physiological cost index and decreased walking speed during walking on uneven surfaces (i.e., placing small strips of wood) (Burridge et al., 2007). Therefore, unlike patients with an incomplete spinal injury and cerebral palsy, how stroke patients adapt to an uneven surface by stepping on the PS during gait remains unclear. Clarifying how poststroke patients adapt to uneven surfaces by step and whether the adaptability to uneven surfaces by stepping on the PS is related to walking activity may be important for understanding the mechanism of the lack of real-world walking activity.

The purpose of this study was to examine stepping patterns during gait on uneven surfaces in poststroke patients and their relationship with real-world walking activity. As compensatory movements such as pelvic hiking and circumduction were used even when walking on level ground (Kerrigan et al., 2000), they may be applied even more owing to the need to take appropriate steps when walking on uneven surfaces. The compensatory movements contributed to energy cost during gait in poststroke patients, which was related to participation in the community (Chen et al., 2005; Stoquart et al., 2012; Franceschini et al., 2013). Postural orientation and dynamic equilibrium, which need balance control, use visual, vestibular, and somatosensory (cutaneous and proprioceptive) sensory information (Horak and Macpherson, 1996). The multiple sensory input is integrated and provides a coherent interpretation of the postural orientation and dynamic equilibrium. The information was available for comparison with an internal body model that maintains balance (Horak and Macpherson, 1996). The pathological dysfunction in the sensory-motor circuitry contributes to the loss of balance function (Darbin et al., 2016; Kuramatsu et al., 2021). However, the compensatory mechanism for somatosensory conditions by visual information completes sit-to-stand in patients after stroke (Kuramatsu et al., 2020). Like sit-to-stand, patients after stroke would be able to adapt gait function on an uneven surface. We hypothesized that the adaptability to uneven surfaces by shorter step length and time with compensatory movement is related to increased real-world walking activity in poststroke patients.

## Materials and Methods

### Subjects

In total, 14 poststroke patients (11 men and 3 women) and 12 healthy controls (8 men and 4 women) of comparable ages and anthropometric characteristics participated in this study (Table 1). The inclusion criteria for poststroke patients were (1) ability to walk without a cane on artificial grass with an approximately 2-cm leaf length over a distance of at least 7 m; (2) a gait speed of > 60 cm/s during walking on an even surface based on the result of a previous study that at the slowest walking speeds (30–50 cm/s), the waist-positioned accelerometer recorded 0 step for approximately 48% of the walking trials; (3) the ability to follow verbal commands; and (4) a poststroke duration of at least a year. The inclusion criterion for controls was subjects without neurological lesions. All the participants were excluded if they had (1) unstable medical conditions, (2) major orthopedic surgery or an actual orthopedic condition interfering with locomotion, and (3) higher brain dysfunction, which skewed the measurements. The nine patients and four healthy controls were treated with blood pressure medication. The participants provided their written and informed consent prior to the start of the experimental session. This study was approved by our institutional review board.

**TABLE 1:** *^a^*Values are means ± standard deviations. *^b^*Anterior cerebral artery, ACA; Middle cerebral artery, MCA. *^c^*The number of subjects who had lacunar infarct lesions (i.e., corona radiata, internal capsule, and both corona radiata and internal capsule) was 3, 2, and 1.

Characteristic	Patients	Controls
N	14	12
Gender (Male/Female)	11/3	8/4
Age (years)^a^	59.3 ± 8.9	59.5 ± 14.6
Height (cm)^a^	169 ± 5.56	163.1 ± 9.15
Weight (kg)^a^	68.1 ± 8.64	62.6 ± 11.49
Diagnosis (Hemorrhage/Infarct)	6/8	
**Type of Hemorrhage**		
Putamen/Thalamus/Subparietal lobe	4/1/1	
**Vascular territory of Infarct**		
ACA infarct^b^/MCA infarct^b^/lucnarinfarct^c^	1/1/6	
Paretic side (Left/Right)	4/10	
Time since stroke (months)^a^	70.7 ± 53.5	
**SIAS motor function (0/1/2/3/4/5)**		
Ankle joint	0/0/0/3/9/2	
Knee joint	0/0/1/8/5/0	
Hip joint	2/1/1/5/5/0	

### Gait Assessment

**FIGURE 1:**
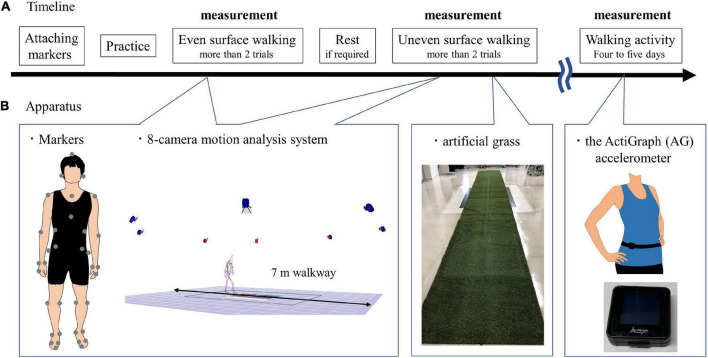
Experimental paradigm. **(A)** Timeline. Even surface walking was completed first by all the participants; uneven surface walking was second after attaching markers. The participants were allowed to rest between the trials if required. After the gait assessment, the subjects wore the ActiGraph (AG) accelerometer (GT9X Link, ActiGraph LLC, Pensacola, FL, United States) around the waist at the height of the iliac crest on the right side in healthy controls and on the non-PS in poststroke patients; the device was held in place by an elastic belt. The AG accelerometer was worn for at least 4 days and at most 5 days. **(B)** Apparatus. Forty markers were attached to 13 segments; a 7 m gait on the even surface and uneven surface was measured using an 8-camera motion analysis system (MAC 3D, Motion Analysis Corporation, Santa Rosa, CA, United States). The artificial grass surface is 7 m long and 1 m wide, with an approximately 2 cm leaf length for uneven surfaces. The size of the AG accelerometer is 35 mm × 35 mm × 10 mm, and its weight is 14 g.

In this study, artificial grass was used during walking in an uneven surface condition. Artificial grass is portable and useful instrumentation to assess walking on uneven surfaces in hospitals. Moreover, the use of artificial grass when walking can change the gait spatiotemporal parameters in patients with incomplete spinal injuries and healthy controls compared with when artificial grass is not used (i.e., even surfaces) (Promkeaw et al., 2019). This study applied the following two surface conditions: even surface in the laboratory and artificial grass surface of 7 m long and 1 m wide, with an approximately 2-cm leaf length for uneven surfaces (Figure 1). Even surface walking was completed first by all the participants. The leaf length used in this study was shorter than that in the previous study (Promkeaw et al., 2019). Although the patients with spinal cord injury in the previous study were allowed to use their customary walking devices (Promkeaw et al., 2019), the poststroke patients in this study were not allowed to be assisted by anyone and to use a cane during walking because their gait parameters were compared with those of the healthy controls, who did not use canes. All of the participants walked over the two surfaces with shoes and orthoses, which they usually wore, at a self-selected speed for more than two trials over each surface. The participants were allowed to rest between the trials, if required. Before the measurement, the participants familiarized themselves with walking over the two surfaces. Forty markers were attached to 13 segments composed of the head, torso, upper arms, forearms, pelvis, thighs, shanks, and feet based on the anthropometric data reported by Okada (Okada et al., 1996; Table 2). Whole-body motion data were collected at 120 Hz by using an 8-camera motion analysis system (MAC 3D, Motion Analysis Corporation, Santa Rosa, CA, United States). The three-dimensional coordinates were smoothed using a bidirectional fourth-order Butterworth lowpass filter with a cutoff frequency of 6 Hz. We detected the gait event (i.e., heel strike and toe off) using the horizontal sacral-heel distance (French et al., 2020). Spatiotemporal parameters were calculated according to the representative method (Butler et al., 2006). The kinematic data for each joint in the lower extremities were calculated using a joint coordinate system (Robertson et al., 2013). The representative parameters of joint angle in the lower limb in the sagittal plane during gait were determined based on the findings of a previous study (Benedetti et al., 1998). The representative parameters related to compensatory strategies in the paretic swing phase were calculated as the segment angle in the global coordination system (Kerrigan et al., 2000). Leg circumduction is the maximum thigh abduction angle in the paretic swing phase. In addition, the magnitude of the symmetry of step length and swing time was calculated as the ratio of the left values to the right values, with the larger value in the numerator (irrespective of the PS) (Patterson et al., 2010). These parameters were calculated using the customized MATLAB software (Mathworks Inc., Natick, MA, United States). The results were the average of more than five strides in successful trials.

**TABLE 2:** Placement of markers on the body.

Segment	Placement of markers
Head	Top of head and both ears, and the spinous process of the 7th cervical vertebrae
Trunk	Spinous process of the 7th cervical vertebrae, spinous process of the 10th thoracic vertebrae, jugular notch where the clavicles meet the sternum, xiphoid process of the sternum, and the position in the middle of the right scapula
Upper arm	Both acromions and both lateral epicondyles of elbow
Forearm	Both lateral epicondyles of the elbow and both styloid processes of the ulna and radius
Pelvis	Both anterior superior iliac spines and both posterior superior iliac spines
Thigh	Both greater trochanters and both lateral and medial epicondyles of knee
Shank	Both lateral epicondyles of knee and both lateral and medial malleolus
Foot	Both the first and fifth metatarsal heads, both lateral and medial malleolus, and both calcaneus

### Walking Activity

After the gait assessment, the subjects wore the ActiGraph (AG) accelerometer (GT9X Link, ActiGraph LLC, Pensacola, FL, United States) around the waist at the height of the iliac crest on the right side in the healthy controls and on the non-PS in the poststroke patients, which was held in place by an elastic belt. The AG accelerometer recorded the three axes of acceleration at 30 Hz by using a proprietary algorithm to calculate step counts with a low-frequency extension (LFE) filter. We used the number of steps per day to evaluate walking activity. The participants were asked to wear the device while awake and to take it off before swimming or bathing. In this study, the AG accelerometer was worn for 14 ± 3 h on a single day by the healthy controls and for 13 ± 3 h for at least 4 days and at most 5 days.

Some subjects felt uncomfortable when they wore the AG accelerometer at the ankle for a day; thus, in this study, the subjects wore the AG accelerometer at the waist. However, a previous study demonstrated that the AG accelerometer produced the most accurate step count in poststroke patients when worn at the non-paretic ankle without LFE but not when worn at the waist (Campos et al., 2018). Therefore, before measurement of the number of steps per day, we evaluated its validity using the AG accelerometer. The subjects with AG walked at their self-selected comfortable pace for 1 min on a 30-m walkway on an even surface two times. The number of walking steps taken within 1 min was recorded using the AG accelerometer and counted by the physical therapist. Agreement in the number of 1-min walking steps calculated using the AG condition and the number of the steps counted by the physical therapist was determined using the Shrout-Fleiss intraclass correlation coefficient [ICC (3,1)] and served as the gold standard. The ICC (3,1) in the healthy controls and patients were 0.48 (*p* = 0.047) and 0.88 (*p* < 0.001). Moreover, the total number of steps counted using the AG accelerometer for 1 min was compared with that counted by the physical therapist, using paired *t*-tests for the data of both groups. Our results showed no significant differences in any of the parameters between the two groups.

The walking activity of 5,000–7,499 steps/day was categorized as least limited community ambulation, and walking activity ≥7,500 was categorized as unlimited community ambulation (Fulk et al., 2017).

### Statistical Analysis

Anthropometric characteristics and walking activity (number of steps/day) were compared between the patients and the healthy controls using unpaired Student’s *t*-tests. Paretic and non-paretic sides in patients and the left side [LS] in healthy controls were compared to examine the effects of the groups and surfaces on gait-related parameters; also, the conditions between the even and uneven surfaces were compared using two-way analysis of variance followed by the Bonferroni *post hoc* test. Moreover, differences in gait parameters between the even and artificial grass surfaces were calculated to determine how the subjects adapted to the artificial grass surface while walking. We examined the association between the differences in surface and walking activity (number of steps per day) using the Pearson product-moment and Spearman’s rank correlation coefficients if the parameter was non-normally distributed. A *p*-value of 0.05 was set as the criterion for statistical significance. Partial beta squared (_*p*_^2^) and Cohen’s *d* were calculated as estimates of the effect size. The statistical analyses were performed using a statistical software package (SPSS version 24; IBM Corp., Armonk, NY, United States).

## Results

No significant differences in body height and weight were found between the patients and the healthy controls (Table 1). Gait speed, spatiotemporal and kinematic parameters during gait, and the number of steps per day for the patients and controls are presented in Tables 3, 4. In addition, Tables 5, 6 showed the correlations between the number of steps per day and the difference in spatiotemporal and kinematic parameters under the conditions of different surface properties.

**TABLE 3:** The mean and standard deviation of spatiotemporal data in patients after stroke and healthy controls.

	Paretic side		Non-paretic side		Left side in healthy controls		Two-way ANOVA *P*-value		
	Even	Uneven	Even	Uneven	Even	Uneven	Subjects	Surface	Interaction
Gait speed (m/s)	0.94 ± 0.20^c^	0.97 ± 0.19^cd^			1.35 ± 0.20	1.38 ± 0.22^d^	0.000	0.015	0.947
Gait cycle time (s)	1.23 ± 0.15^c^	1.20 ± 0.14^cd^			1.03 ± 0.06^a^	1.02 ± 0.14^*ad*^	0.001	0.005	0.204
Stance time (s)	0.66 ± 0.09^c^	0.64 ± 0.08^cd^	0.71 ± 0.12^c^	0.69 ± 0.11^cd^	0.55 ± 0.04^ab^	0.55 ± 0.05^abd^	<0.001	0.001	0.414
Swing time (s)	0.57 ± 0.07^bc^	0.56 ± 0.08^bcd^	0.51 ± 0.05^a^	0.50 ± 0.05^ad^	0.48 ± 0.03^a^	0.48 ± 0.04^ad^	0.001	0.001	0.503
Step length (m)	0.57 ± 0.08	0.56 ± 0.08	0.51 ± 0.06	0.52 ± 0.06	0.65 ± 0.07	0.65 ± 0.09	<0.001	0.149	0.237
Stride length (m)	1.12 ± 0.15^c^	1.13 ± 0.14^c^			1.38 ± 0.18^a^	1.39 ± 0.20^a^	0.001	0.369	0.846
Step width (m)	0.15 ± 0.04	0.16 ± 0.04^d^			0.13 ± 0.03	0.15 ± 0.03^d^	0.284	0.000	0.345
Stance time symmetry	1.09 ± 0.06^c^	1.08 ± 0.07^c^			1.03 ± 0.02^a^	1.02 ± 0.0.02^a^	0.003	0.589	0.996
Swing time symmetry	1.11 ± 0.09^c^	1.11 ± 0.10^c^			1.03 ± 0.03^a^	1.03 ± 0.02^a^	0.004	0.661	0.9.34

*^a^Significantly different from the paretic side at p < 0.05.*

*^b^Significantly different from non-paretic side at p < 0.05.*

*^c^Significantly different from healthy controls at p < 0.05.*

*^d^Significantly different from even surface at p < 0.05.*

**TABLE 4:** The mean and standard deviation of angles in lower limb in patients after stroke and healthy controls.

	Paretic side		Non-paretic side		Left side in healthy controls		Two-way ANOVA *P*-value		
	Even	Uneven	Even	Uneven	Even	Uneven	Subjects	Surface	Interaction
Ankle plantarflexion in early stance	5.4 ± 5.7	5.4 ± 5.9	3.9 ± 4.0	3.5 ± 3.7	3.2 ± 2.9	2.9 ± 2.9	0.374	0.301	0.679
Ankle dorsiflexion in stance	−11.4 ± 8.2	−11.0 ± 9.2	−17.4 ± 5.7	−16.7 ± 6.0	−13.9 ± 7.0	−14.7 ± 6.4	0.110	0.834	0.279
Ankle plantarflexion at toe-off	−3.1 ± 8.5	−2.6 ± 8.8	−4.3 ± 8.1	−4.1 ± 7.9	−4.1 ± 7.5	−4.2 ± 7.7	0.885	0.476	0.629
Ankle dorsiflexion in swing	−4.8 ± 7.3	−4.4 ± 8.2	−8.5 ± 5.1	−8.1 ± 4.9	−6.8 ± 5.2	−7.1 ± 4.8	0.270	0.666	0.547
Knee flexion in early stance	−10.7 ± 8.7	−10.8 ± 9.3	−12.2 ± 8.0	−12.7 ± 7.9	−5.8 ± 4.1	−7.1 ± 3.7	0.123	0.052	0.274
Knee extension in stance	−1.7 ± 9.8	−1.8 ± 10.2	−2.1 ± 6.5	−1.8 ± 6.3	−3.3 ± 6.2	−4.6 ± 3.6	0.728	0.458	0.355
Knee flexion at toe-off	−25.1 ± 7.0^b^	−24.4 ± 7.6^b^	−31.2 ± 5.6^a^	−30.7 ± 5.3^a^	−24.0 ± 7.6	−25.2 ± 3.7	0.012	0.954	0.399
Knee flexion in swing	−46.2 ± 12.8^bc^	−48.4 ± 12.5^bcd^	−66.1 ± 5.1^a^	−67.5 ± 5.5^ad^	−61.0 ± 13.1^a^	−65.8 ± 2.4^ad^	<0.001	0.011	0.404
Hip flexion at foot contact	−29.0 ± 4.2^b^	−29.5 ± 4.5^bd^	−35.1 ± 6.5^a^	−36.0 ± 6.7^ad^	−32.3 ± 6.8	−34.0 ± 5.0^d^	0.015	0.042	0.537
Hip extension in stance	−0.2 ± 5.4^bc^	−0.1 ± 5.8^bc^	5.8 ± 4.6^a^	6.3 ± 3.8^a^	5.0 ± 5.0^a^	6.3 ± 3.4^a^	0.004	0.319	0.182
Hip flexion at foot off	−4.6 ± 6.1^bc^	−4.4 ± 6.3^bc^	1.6 ± 4.4^a^	1.4 ± 4.9^a^	2.9 ± 5.7^a^	4.4 ± 3.7^a^	0.001	0.245	0.246
Hip flexion during swing	−35.0 ± 2.7^b^	−36.3 ± 2.4^bd^	−40.0 ± 5.5^a^	−41.4 ± 5.7^ad^	−36.7 ± 5.6	−39.0 ± 4.6^d^	0.018	0.000	0.392
Maximum toe clearance (cm)	11.6 ± 2.4	12.4 ± 2.4^d^			13.3 ± 1.8	14.0 ± 1.6^d^	0.057	0.000	0.679
**Compensatory movements (degrees)**									
Thorax anterior tilt in the sagittal plane	−4.5 ± 2.5	−5.5 ± 2.7^d^			−4.8 ± 2.5	−5.7 ± 2.4^d^	0.797	0.000	0.831
Pelvic anterior tilt in the sagittal plane	−9.1 ± 4.0	−9.7 ± 4.4^d^			−10.8 ± 3.4	−11.1 ± 4.0^d^	0.338	0.018	0.428
Circumduction of lower limb	2.1 ± 4.5	2.0 ± 5.0			0.8 ± 2.4	1.2 ± 2.4	0.020	0.263	0.096

*^a^Significantly different from paretic side at p < 0.05.*

*^b^Significantly different from non-paretic side at p < 0.05.*

*^c^Significantly different from healthy controls at p < 0.05.*

*^d^Significantly different from even surface at p < 0.05.*

**TABLE 5:** The correlations between walking activity and surface difference in spatiotemporal parameters.

	Correlation	
	Paretic side	Left side in healthy controls
Gait speed (m/s)	0.49	–0.04
Gait cycle time (s)	–0.13	–0.32
Stance time (s)	–0.04	–0.24
Swing time (s)	–0.10	–0.42
Step length (m)	0.65^a^	0.02
Stride length (m)	0.48	0.07
Step width (m)	–0.06	0.68^a^
Symmetry step length	0.03	0.46
Symmetry stance time	–0.11	0.53
Symmetry swing time	0.02	0.55

*^a^p < 0.05.*

**TABLE 6:** The correlations between walking activity and surface difference in kinematic parameters.

	Correlation		
	Paretic side	Non-paretic side	Left side in healthy controls
**Peak angle**			
Ankle plantarflexion in early stance	0.24	0.18	–0.18
Ankle dorsiflexion in stance	0.10	0.28	–0.03
Ankle plantarflexion at toe-off	0.11	0.68^b^	–0.21
Ankle dorsiflexion in swing	0.16	0.58^a^	0.03
Knee flexion in early stance	0.31	0.20	0.78^b^
Knee extension in stance	0.31	0.59^a^	0.28
Knee flexion at toe-off	0.24	0.24	–0.00
Knee flexion in swing	–0.11	–0.31	–0.40
Hip flexion at foot contact	−0.69^b^	–0.09	0.45
Hip extension in stance	0.25	0.35	–0.10
Hip flexion at foot off	0.32	0.18	–0.08
Hip flexion during swing	–0.45	–0.14	0.20
**Compensatory movements during swing (degrees)**			
Pelvic elevation on the PS in the frontal plane	–0.07		−0.71^b^
Thorax anterior tilt in the sagittal plane	–0.13		0.05
Pelvic anterior tilt in the sagittal plane	–0.33		0.35
Circumduction	0.43		0.38

*^a^p < 0.05;*

*^b^p < 0.01.*

### Walking Activity

The number of steps per day of the poststroke patients (10,118.0 ± 4,550.2 steps/day) was significantly less than that of the healthy controls (15,376.6 ± 5,440.1 steps/day; *p* = 0.013, *d* = 1.06). All the healthy controls and 78.6% of the poststroke patients were categorized as unlimited ambulators. In addition, 21.4% of the poststroke patients were categorized as least limited community ambulators.

### Spatiotemporal Parameters

Gait speed on the even surface in the patients with hemiparesis and healthy controls was faster than 0.71 m/s. There were three patients with a shorter step length on the PS than step length on the non-PS when walking on the even surface. When walking on the uneven surface, in addition to the three patients, one patient had a longer step length on the non-PS.

Significant effects for group factor on gait speed [F_(1,24)_ = 27.02, *p* < 0.001, η_*p*_^2^ = 0.53], gait cycle time [*F*_(1,24)_ = 15.38, *p* = 0.001, η_*p*_^2^ = 0.39], stance [F_(2,37)_ = 10.45, *p* < 0.001, η_*p*_^2^ = 0.36] and swing times [F_(2,37)_ = 7.99, *p* = 0.001, η_*p*_^2^ = 0.30], step [F_(2,37)_ = 11.46, *p* < 0.001, η_*p*_^2^ = 0.38], and stride length [F_(1,24)_ = 15.57, *p* = 0.001, η_*p*_^2^ = 0.39] were observed. Gait speed, gait cycle time, and stride length in the patients were slower (0.95 ± 0.19 m/s vs. 1.37 ± 0.21 m/s), longer (1.21 ± 0.15 s vs. 1.03 ± 0.07 s), and shorter (1.13 ± 0.14 m vs. 1.38 ± 0.19 m) than those in the healthy controls. The stance times on the PS (*p* = 0.019, *d* = 1.49) and non-PS (*p* < 0.001, *d* = 1.74) were significantly longer than that on the LS in the healthy controls, although the step lengths on the PS (*p* = 0.012, *d* = 1.13) and non-PS (*p* < 0.001, *d* = 1.96) were shorter. Swing time on the PS was longer than that on the non-PS (*p* = 0.034, *d* = 0.91) and LS (*p* = 0.001, *d* = 1.42).

Significant effects for surface factor on gait speed [F_(1,24)_ = 6.83, *p* = 0.015, η_*p*_^2^ = 0.22], gait cycle time [F_(1,24)_ = 15.38, *p* = 0.005, η_*p*_^2^ = 0.28], stance [F_(1,37)_ = 13.24, *p* = 0.001, η_*p*_^2^ = 0.26] and swing times [F_(1,37)_ = 12.45, *p* = 0.001, η_*p*_^2^ = 0.25], and step width [F_(1,24)_ = 16.50, *p* < 0.001, η_*p*_^2^ = 0.41] were observed but not on stride and step length. The temporal parameters on the artificial grass surface were smaller than those on the even surface, although gait speed was faster on the artificial grass surface (1.13 ± 0.29 m/s vs. 1.16 ± 0.29 m/s). The step width on the artificial grass surface was larger than that on the even surface (0.14 ± 0.04 m vs. 0.15 ± 0.03 m).

**FIGURE 2:**
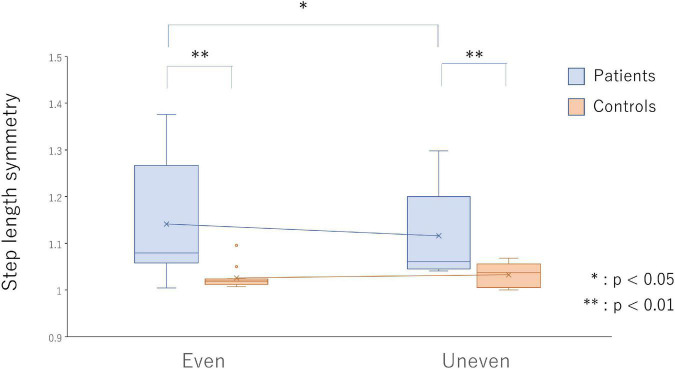
Step length symmetry on the even surface (bars on the left side) and uneven surface (bars on the right side) in the poststroke patients (light blue bars) and the healthy controls (light red bars). *p < 0.05 and ^**^p < 0.01 indicate statistical significance.

Significant interactions were found between the group and the surface in terms of step length symmetry [F_(1,24)_ = 5.44, *p* = 0.028, η_*p*_^2^ = 0.19]. In the poststroke patients, gait on the artificial grass surface showed significantly greater symmetry in step length (*p* = 0.013, *d* = 0.24) but not in the healthy controls (Figure 2).

### Kinematic Parameters

Significant effects for group factor on knee flexion at foot off [F_(2,37)_ = 4.97, *p* = 0.012, η_*p*_^2^ = 0.21], maximum knee flexion during swing [F_(2,37)_ = 18.46, *p* < 0.001, η_*p*_^2^ = 0.50], hip flexion at initial contact [F_(2,37)_ = 4.70, *p* = 0.015, η_*p*_^2^ = 0.20], maximum hip extension in stance [F_(2,37)_ = 6.58, *p* = 0.004, η_*p*_^2^ = 0.26], hip flexion at foot off [F_(2,37)_ = 9.06, *p* = 0.001, η_*p*_^2^ = 0.33], and maximum hip flexion during swing [F_(2,37)_ = 4.52, *p* = 0.018, η_*p*_^2^ = 0.20] were observed. Knee flexion at foot off was highest on the non-PS, and hip flexion at foot off was highest on the PS. The maximum knee flexion angles during swing and hip extension in stance were lowest on the PS. Hip flexion at initial contact (*p* = 0.013, *d* = 1.15) and during swing (*p* = 0.014, *d* = 1.18) on the PS was significantly lower than that on non-PS.

Significant effects for surface factor on maximum knee [F_(1,37)_ = 7.20, *p* = 0.011, η_*p*_^2^ = 0.16] and hip flexion [F_(1,37)_ = 28.14, *p* < 0.001, η_*p*_^2^ = 0.43], and thoracic [F_(1,24)_ = 45.60, *p* = 0.001, η_*p*_^2^ = 0.66] and pelvic anterior tilts [F_(1,24)_ = 6.43, *p* = 0.018, η_*p*_^2^ = 0.21] in the sagittal plane during swing phase, hip flexion at initial contact [F_(1,37)_ = 4.42, *p* = 0.042, η_*p*_^2^ = 0.11], and maximum toe clearance [F_(1,24)_ = 29.66, *p* < 0.001, η_*p*_^2^ = 0.55] were observed. All the angles on the artificial grass surface in the patients and healthy controls were larger than those on the even surface.

**FIGURE 3:**
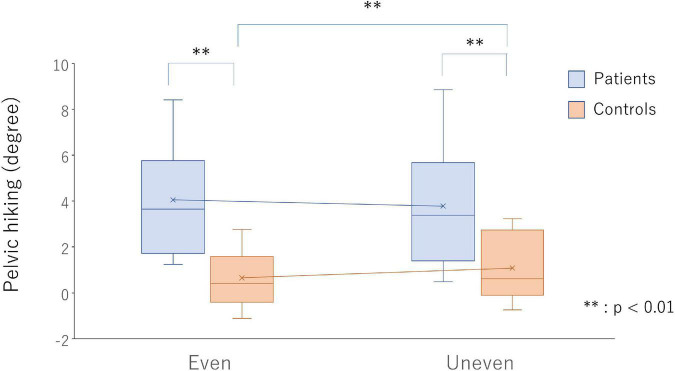
Pelvic hiking on the even surface (bars on the left side) and uneven surface (bars on the right side) in the poststroke patients (light blue bars) and healthy controls (light red bars). ^**^ *p* < 0.01 indicate statistical significance.

A significant interaction was observed in terms of pelvic tilt in the frontal plane during swing [F_(1,24)_ = 12.63, *p* = 0.002, η_*p*_^2^ = 0.35]. The maximum pelvic elevation on PS in the frontal plane during a swing in the poststroke patients increased as compared with that in the healthy controls on both the even (*p* < 0.001, *d* = 1.74) and artificial grass surfaces (*p* = 0.003, *d* = 1.28). In the poststroke patients, decreased maximum pelvic elevation on the PS during swing (*p* = 0.055, *d* = 0.11) was observed during gait on the artificial grass surface as compared with the even surface, but the increased maximum pelvic elevation was observed in the healthy controls (*p* = 0.007, *d* = 0.31) (Figure 3).

### Relationship Between Walking Activity and Difference in Gait-Related Parameters

**FIGURE 4:**
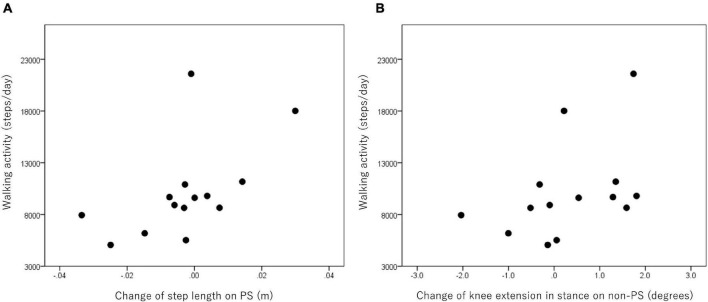
Relationship between walking activity and changes in step length on the paretic side (PS) **(A)** and knee extension in stance on the non-PS **(B)** in the poststroke patients.

In the patients with hemiparesis, the difference in step length on the PS significantly and positively correlated with the number of steps per day (*r* = 0.65, *p* = 0.012; Figure 4). The difference in maximum knee extension in the stance phase (*r* = 0.59, *p* = 0.026; Figure 4) and ankle plantarflexion at foot off on the non-PS (*r* = 0.68, *p* = 0.008) were also significantly and positively associated with the number of steps per day, but the maximum ankle dorsiflexion during swing was negatively associated (*r* = −0.58, *p* = 0.029). However, no significant relationship was found between the difference in compensatory movements during the swing and the number of steps per day.

In the healthy controls, the difference in step width significantly and positively correlated with the numbers of steps per day (*r* = 0.68, *p* = 0.015). The difference in the maximum knee extension at initial contact was also associated with the number of steps per day (*r* = 0.76, *p* = 0.004).

## Discussion

Our findings showed a more symmetrical step length pattern and decreased pelvic hiking (i.e., pelvic elevation on PS in the frontal plane) in the swing phase, which indicates reduced compensatory movement, during walking on the artificial grass surface in the community ambulators after stroke. In addition, the changes in paretic step length, non-paretic knee extension, and ankle plantarflexion in the stance phase, and paretic hip flexion at initial contact were related to walking activity in the community ambulators after stroke. Contrary to our hypothesis, the walking adaptability to the artificial grass surface by increasing the paretic step length with compensation movement by the non-paretic lower limb during gait may increase walking activity in the community ambulators after stroke.

Both the poststroke patients and healthy controls adjusted with reduced gait cycle and swing times (i.e., step time) on both sides and increased gait speed but without changing the step length when walking on the artificial grass surface. The present results on step length and gait speed were contrary to our hypothesis. In the previous study on the lateralization of stroke in the chronic phase, right hemisphere damage was related to a slower walk and more asymmetry in single stance time (Chen et al., 2014). Guye et al. (2003) found a more extensive and stronger connected network in the dominant left hemisphere, carrying out the processing of sensory-motor data. This fact means that the extensive and strong connected network in the dominant left hemisphere may easily compensate for one damaged network component. Ten out of fourteen patients in this study had left hemisphere damage. Therefore, the patients who participated may change the parameter regarding rhythm with a change from an even surface to a short leaf grass surface during gait. In this study, unlike the previous study, the artificial grass surface did not cause a considerable cautious gait pattern (i.e., reduced gait speed, cadence, and step length) (Paysant et al., 2006; Menant et al., 2009; Promkeaw et al., 2019). Paysant et al. (2006) showed that the gait spatiotemporal parameters differed between an asphalt surface and grass surface with a leaf length of 12–20 cm in the healthy controls and amputees but not in the grass surface with a leaf length < 2–4 cm, which is similar to the length of the artificial grass used in this study. When standing on foam, information from both joint and cutaneous mechanoreceptors of the sole is less reliable, increasing body movement (Chiang and Wu, 1997; Fransson et al., 2007). To decrease tripping and slipping, gait on foam resulted in increased step length, width, and time compared with walking on a level surface (Maclellan and Patla, 2006). The short leaf grass, unlike foam, might produce sufficient information from both joint and cutaneous mechanoreceptors of the sole because short leaf grass is not soft like foam. Therefore, this study shows that with the short leaf length, gait stability on the artificial grass may be easier to maintain and may not need a shorter step length. Hsiao et al. (2016) demonstrated that the poststroke patients increased forward propulsion on the non-PS as the primary mechanism to increase gait speed. Considering the findings of the previous study, increased gait speed in the condition of an artificial grass surface may be attributed to the slip-resistant surface (i.e., artificial grass with short leaf has high friction property) on which subjects could generate propulsion. Moreover, the mean gait speed of the patients in this study (0.94 m/s) was faster than that of the patients categorized as a high-functioning group who can increase hip flexion power and ankle plantarflexion power on the PS to increase gait speed and reduce stride time (Jonkers et al., 2009; Sekiguchi et al., 2012). Therefore, the poststroke patients with high function in this study may be able to decrease step time and increase gait speed during gait on the artificial grass with a short leaf length compared with the healthy controls.

Only the poststroke community ambulators adapted gait on the artificial grass surface by increasing their symmetrical step length. A previous study that examined interlimb cutaneous reflexes demonstrated that in poststroke patients, the somatosensory network related to interlimb coordination between the legs and the arms was preserved, which indicates that the patients may have the ability to increase step length symmetry (Zehr and Loadman, 2012). The unevenness of the artificial grass might provide more somatosensory information from the plantar region than the evenness of the even surface in the patients who can increase their step length symmetry. Moreover, step length asymmetry was associated with propulsion asymmetry in the poststroke patients (Roelker et al., 2019). The slip-resistant surface of the artificial grass with a short leaf length might make propulsion easier to control. Therefore, the patients can increase their step length symmetry when walking on the artificial grass with a short leaf length. As a result, increased step length symmetry, which was related to balance measures (i.e., Berg balance scale), might increase stability when walking on the artificial grass in the poststroke patients (Lewek et al., 2014).

There were two types of strategies for the paretic step length for an uneven surface in this study. Five patients had a longer paretic step length on the uneven surface than on the even surface. Four of the five patients with a longer paretic step length had a longer non-paretic step length on the uneven surface than on the even surface. However, the other patient had a shorter non-paretic step length on the uneven surface. In the results, three of the five patients with a longer paretic step length on the uneven surface increased step length symmetry. In contrast, nine patients had a shorter paretic step length on the uneven surface than the even surface. Four of the nine patients with the shorter paretic step length had a shorter non-paretic step length on the uneven surface than on the even surface. However, five patients had a longer non-paretic step length on the uneven surface. Additionally, all five patients had a longer paretic step length than non-paretic step length on the even surface. Therefore, the patients with a shorter paretic step length on the uneven surface may maintain or increase the step length symmetry. In fact, seven of the nine patients with a shorter step length on the uneven surface increased step length symmetry. In summary, most patients with a longer paretic step length did not follow the strategy of non-paretic steps that would result in step length asymmetry similar to the patients with a shorter paretic step. Consequently, most patients after stroke with a longer paretic step length and more walking activity, but not all, may not have less step length symmetry on the uneven surface.

The hip and knee flexion angles during a swing on the artificial grass surface increased as compared with those on the even surface in the poststroke patients and healthy controls, consistent with the previous study in patients with multiple sclerosis and cerebral palsy (Böhm et al., 2014; van den Berg et al., 2017). When considered in conjunction with the present results showing increased toe clearance, the increase in hip and knee flexion angles during swing may lead to an increase in foot clearance on the artificial grass surface in both groups. Artificial grass surface was not used in the previous studies that examined toe clearance on the uneven surface (Böhm et al., 2014; van den Berg et al., 2017). In addition to walking on the other uneven surfaces, walking on artificial grass with a short leaf length may be necessary to increase the height of toe clearance as compared with that on the even surface. In line with the previous findings in patients with cerebral palsy (Böhm et al., 2014), greater anterior tilt angles of the pelvic and thorax were found in poststroke patients during the swing phase when walking on an artificial grass surface. The present findings show that decreased pelvic hiking during gait on the artificial grass surface was found only in the poststroke patient. Increased pelvic and thoracic anterior tilt angles and decreased pelvic hiking may lower the height of the center of mass during gait in poststroke patients, as shown in previous studies (Chen et al., 2005; Saha et al., 2008). During gait on the artificial grass with a short leaf length, control of the trunk posture and increase in toe clearance may be necessary for maintaining dynamic stability, but the changes in the gait spatiotemporal parameters such as caution gait (i.e., short step length and slow gait speed) may not be necessary because maintaining gait on artificial grass with a short leaf length is an easier task.

The change in paretic step length, but not step width, from the even surface to the artificial grass was related to walking activity in the poststroke patients. The walking activity of the healthy controls was related to the increase in the step width when walking on the artificial grass. Like the healthy controls, the patients may have difficulty controlling their step width because of deficit of control in the frontal plane by the hip and ankle joints (Kim and Eng, 2004; Neckel et al., 2008; Sánchez et al., 2018; Hsiao et al., 2020). The healthy controls with increased walking activity increased their step width on the artificial grass, which increased the base of support like in cautious gait (McAndrew Young and Dingwell, 2012). Although maintaining gait on the artificial grass with a short leaf length is an easier task, high community walking activity may require adaptation on the artificial grass with a short leaf length not only by increasing step length on the PS in the poststroke patient but also by increasing the step width in the healthy controls to increase the base of support and maintain stability control. In addition, the walking activity of the poststroke patients correlated with the changes in the maximum knee extension in the stance phase and ankle plantarflexion at foot off on the non-PS and that of the step length on the PS. Consistent with the kinematic changes in the responders of walking activity after training (Ardestani et al., 2019), the kinematic change in the lower limb on the non-PS was associated with walking activity in patients. The poststroke patients with increased walking activity may also need the compensatory movement of the leg on the non-PS to increase their step length on the PS, like responders of walking activity after training.

This study has several limitations. First, the least limited and unlimited community ambulators participated in the study but not most limited and household ambulators. Therefore, the question of whether walking adaptability to the artificial grass surface was related to walking activity in most limited and household ambulators after stroke remains unclear. Second, kinetic data were not collected. Future studies should clarify whether power generated at the knee and hip joints during gait on the artificial grass surface is more needed on the non-PS than on the even surface and the energy cost during gait on the artificial grass surface in poststroke patients (Farris et al., 2015). Third, this study used only one kind of uneven surface (i.e., an artificial grass). Previous studies on gait on uneven surfaces used surfaces different from artificial grass. Several surfaces (i.e., foam, sand, railroad ballast, artificial grass, and artificial pebble surface) caused differences in the results of the step parameters (Maclellan and Patla, 2006; Wade et al., 2010; van den Berg et al., 2017; Promkeaw et al., 2019). Therefore, our findings are restricted to poststroke patients categorized as least limited and unlimited community ambulators and walking on short leaf artificial grass. Fourth, the intraclass correlation coefficient between the number of steps calculated from the accelerometer and the number of steps counted by the therapist was low in the healthy controls. In the healthy controls, the difference between the number of steps counted by the therapist and the number of steps calculated from the accelerometer was extremely larger than other subjects (i.e., the maximum difference in the healthy control: 43 steps vs. the other healthy controls: 5 steps). The result demonstrated the limitation of measuring the step count with an accelerometer. One factor that prevented AG from accurately detecting steps could be using an LFE filter in the healthy controls, as shown in a previous study (Campos et al., 2018). However, there is no difference in the main result between the data in all healthy controls and that excluding the healthy control No. 3 [i.e., also in the eleven healthy controls excluding No. 3, the difference in step width significantly and positively correlated with the numbers of steps per day (*r* = 0.69, *p* = 0.015)].

## Data Availability Statement

The raw data supporting the conclusion of this article will be made available by the authors, without undue reservation.

## Ethics Statement

The studies involving human participants were reviewed and approved by The Research Ethics Committees at Tohoku University Graduate School of Medicine. The patients/participants provided their written informed consent to participate in this study.

## Author Contributions

YS and KH conceived and designed the experiments and collected and analyzed the data. YS wrote the manuscript. YS, KH, and S-II revised the manuscript critically. All authors read and approved the final manuscript.

## Conflict of Interest

The authors declare that the research was conducted in the absence of any commercial or financial relationships that could be construed as a potential conflict of interest.

## Publisher’s Note

All claims expressed in this article are solely those of the authors and do not necessarily represent those of their affiliated organizations, or those of the publisher, the editors and the reviewers. Any product that may be evaluated in this article, or claim that may be made by its manufacturer, is not guaranteed or endorsed by the publisher.
